# Has the Dental Profession Taken Leave of Its Senses?

**Published:** 1917-05

**Authors:** C. N. Johnson


### HAS THE DENTAL PROFESSION TAKEN LEAVE OF ITS SENSES?

BY C. N. JOHNSON, D.D.S.

The foregoing query is naturally suggested by some of the statements appearing from time to time in our dental magazines. If we followed the advice of all the writers of the day, we would scarce practice dentistry at all, but would meekly stand when a patient applies for relief, and in a ''holier-than-thou'' attitude declare solemnly that it was no longer authorized procedure to do many of the things by which the profession had been serving the people for years.

We would lay aside our gold pluggers; we would banish bridgework, particularly fixed bridges; we would never—no, never—use arsenic for destroying pulps; we would even look askance at pressure anesthesia for this purpose; we would never fill pulp canals without checking up our work every few minutes with the X-ray; we would never consider a pulpless tooth safe from focal infection in which the X-ray failed to show the filling beyond the end of the root; we would never leave a tooth in the mouth if the X-ray showed the slightest trace of rarefaction at the root end; we would never attempt to preserve a tooth if it had once been abscessed, etc., etc. With what result? Simply forceps and artificial dentures—and subsequent profanity from our patients.

Most of these writers are perfectly conscientious, and what is more, most of them have seen the light. The trouble is that they have concentrated their vision on only a few rays instead of taking in the whole glorious horizon. Too often in this as in every profession men become permeated with an idea and shut their eyes to every fact which does not fit into that idea.

Let us briefly examine some of the recent pronouncements. It goes without saying that the introduction of the gold inlay was a blessing to the profession and to humanity, but the man who practices without using his gold pluggers is not in every case doing the best service for his patients. Bridgework and all the principles which should govern its use have been outrageously abused, and much harm has been done thereby; but this does not alter the fact that bridges—fixed bridges—from a few teeth to many, have in numberless cases done excellent service and given much satisfaction to patients, adding comfort and years to their lives. Fixed bridges have been known to last more than twenty years, remaining firm and satisfactory for that time, and if the principle of the fixed bridge were wholly wrong, there would never have been so many of these successful cases. This does not mean that a better plan should not be welcomed if it presents itself, but too frequently in the past these "better plans" have not turned out to be better. Nor does it mean that we should not put more discriminating thought and better judgment into all of our bridgework, and profit by the mistakes of the past. If we do, this bridgework will still remain a blessing.

Arsenic is a dangerous drug. Everyone admits that, or should admit it. Considering the manner in which it has been used in the past, the marvel is that it had done as little harm as it has. It has been "slopped about promiscuous" by all sorts of slipshod methods, and has naturally left some sad tales in its wake. But used judiciously, it has a definite and legitimate place in pulp destruction, else its general employment for the past half a century would have long since relegated it to oblivion. Men do not go on blundering in a practice like that for more than fifty years. When something better and safer for destroying the pulp is introduced, the profession will be glad to welcome it. The introduction of pressure anesthesia promised at first to solve the problem, but it turned out a failure in some cases, and in many hands; and now there is a cry against it for various reasons—the danger of forcing infection into the apical region, the resultant soreness in some instances, its unreliability in others, and the difficulty experienced at times in confining the drug and forcing it into the pulp. And yet the fact is that for certain cases pressure anesthesia is ideal—the moral of which is that we need different methods to meet different cases.

Those who are today condemning arsenic and pressure anesthesia in a sweeping way and advocating conductive and interosseous anesthesia for the removal of pulps, are promulgating a doctrine which will never stand the test of time or experience. It is a most grotesque and unreasonable way of removing a pulp, to say nothing of the untoward results which are likely to follow in its wake. We need progress and the introduction of new ideas, but we should have a care that the enthusiasm of the new does not lead us into the promulgation of a foolish and dangerous practice.

The X-ray is a most useful agent in pulp canal and other work, but to say that no pulp canal should be filled without checking up with X-ray pictures at frequent intervals is sheer folly. In a paper read before the Chicago Dental Society and published in the April number of the *Dental Review,* Dr. Edmund Noyes made in a few words the sanest statement about pulp canal work that we have recently seen—stripping it of much of the intricacy and mystery which has been thrown around it. It will pay the profession to read that article.

The limits of editorial space will not permit a further consideration of the extremes which men are teaching today, but the advice is hereby given to those in the profession who have been driven into doubt and even into despair by the extravagant statements that are being widely made, that they shall preserve their balance, do the very best they can for their patients always and forever, and refuse to be stampeded out of a legitimate line of practice which their own experience has taught them to be safe and sane.

In a letter to Dr. C. N. Johnson in regard to an editorial published in the *Dental Review,* Dr. L. E. Custer makes the following comment on the above subject:

I want to emphasize your position on the general extraction of those teeth which when rayed show light areas about their root apices. These are not all abscesses—not by a long shot. I happened to be the second dentist to take up the X-ray in dentistry, Dr. E. C. Kells being the first; and after a period of twenty-two years in X-ray work and study of the behavior of teeth showing light areas about the root apices, I am prepared to show that ninety per cent., and perhaps more, of all pulpless teeth show rarefaction about their root apices.

Why is this, and what does it mean? My answer is that nature never intended that a tooth should be pulpless, and no matter how well a pulp canal may be prepared and filled, it still is not a normal pulp canal. The apical foramen and region thereabout is the vital point in the whole proposition, and, unfortunately, the apex is the most difficult to seal in a manner comfortable and non-irritating to nature. The unfilled apex or an apex sealed with a foreign material is an irritant, although it may be slight, to the surrounding tissues. The result is the resorbtion of the bone in the immediate vicinity and its replacement with a new tissue which when completed encysts the end of the root. Now the encysting tissue being devoid of lime salts, shows dark in the skiagraph, and unless one has had considerable experience in the observation of these cases, he will make the common mistake of pronouncing such skiagraph as showing an abscess.

It is a fine how-de-do when a physician at this day, without any dental experience in these things, shall dictate to a dentist, authorizing the extraction of such teeth. That there are abscesses about the roots of pulpless teeth we are not denying, but anyone with X-ray experience can tell the difference between an encysted root and an abscess. The correct interpretation of a skiagraph in dental cases can not be made by a physician unless he has had a long clinical observation in dental practice, and it is not his place to authorize extraction by a mere glance at a skiagraph.—*Dental Review*, for May.

